# Interleukin 12 in the Acute Phase of the Immune Response after Excimer Laser Treatment

**DOI:** 10.3390/jcm12134472

**Published:** 2023-07-04

**Authors:** Mirko Resan, Zeljka Cvejic, Igor Pancevski, Gabriele Thumann, Martina Kropp, Ivo Guber, Dragana Ristic, Danilo Vojvodic, Bojan Pajic

**Affiliations:** 1 Eye Clinic, Military Medical Academy, 11000 Belgrade, Serbia; dr.panchevski@gmail.com (I.P.); dadana25@yahoo.com (D.R.); 2 Faculty of Medicine of the Military Medical Academy, University of Defense, 11000 Belgrade, Serbia; vojvodic.danilo@gmail.com (D.V.); bojan.pajic@orasis.ch (B.P.); 3 Department of Physics, Faculty of Sciences, University of Novi Sad, 21000 Novi Sad, Serbia; zeljka.cvejic@df.uns.ac.rs; 4 Division of Ophthalmology, Department of Clinical Neurosciences, Geneva University Hospitals, 1205 Geneva, Switzerland; gabriele.thumann@hcuge.ch (G.T.); martina.kropp@unige.ch (M.K.); ivo_guber@hotmail.com (I.G.); 5 Experimental Ophthalmology, University of Geneva, 1205 Geneva, Switzerland; 6 Department for Clinical and Experimental Immunology, Institute for Medical Research, Military Medical Academy, 11000 Belgrade, Serbia; 7 Eye Clinic ORASIS, Swiss Eye Research Foundation, 5734 Reinach, Switzerland

**Keywords:** IL-12, IL-4, IL-10, excimer laser, LASIK, PRK, tears, corneal wound healing

## Abstract

Background and objectives: The aim of the research was to investigate the differences in the concentrations of IL-12, IL-4, IL-10, and IFN-γ in tears after LASIK and PRK procedures. Materials and methods: The study included 68 myopic eyes up to −3.0 D refractive spherical equivalent, divided into two groups: Group 1 LASIK (*n* = 31) and Group 2 PRK (*n* = 37). Three tear samples were taken from each eye: immediately before the procedure (t_0_), 1 h after the procedure (t_1_), and 24 h after the procedure (t_2_). The concentrations of IL-12p70, IL-4, IL-10, and IFN-γ in the tear samples were determined by flow cytometry. Participants were not taking anti-inflammatory therapy 24 h after the procedure. Results: IL-4 levels 1 h after treatment did not differ between LASIK and PRK (*p* = 0.990), while 24 h after PRK there was a significant decrease in IL-4 levels (*p* < 0.05), but not after LASIK (*p* = 0.476). In both the LASIK (*p* < 0.05) and PRK (*p* < 0.05) groups, there is an increase in IL-10 concentrations 1 h after treatment, which persists 24 h after LASIK (*p* < 0.05) but not after PRK (*p* = 0.081). There is an increase in IL-12p70 concentration 1 h after treatment in both the LASIK (*p* < 0.001) and PRK groups (*p* < 0.001). There is also an increase in IL-12p70 concentration 24 h after PRK (*p* < 0.005), but not after LASIK (*p* = 0.775). Conclusions: IL-4 concentration shows a significantly higher value in the LASIK group than in the PRK group after 24 h. IL-10 and IL-12p70 levels increase one hour after surgery in both groups. After 24 h, the IL-10 levels remain elevated in the LASIK group, and the IL-12p70 levels remain elevated in the PRK group. Thus, LASIK and PRK procedures show different inflammatory dynamics.

## 1. Introduction

Interleukin 12 (IL-12) belongs to the type I cytokine family, and the principal cellular sources of this cytokine are macrophages and dendritic cells. Its receptor CD212 is composed of two chains: IL-12Rβ1 and IL-12Rβ2. T cells (Th1 differentiation), NK cells, and T cells (IFN-γ synthesis and increased cytotoxic activity) are the principal cellular targets of IL-12. IL-12 is a covalently linked heterodimer (referred to as IL-12p70) with a light chain, IL-12A (p35), and a heavy chain, IL-12B (p40), which controls its biological activity. IL-12 builds its own cytokine family of heterodimeric cytokines, to which IL-23, IL-27, and IL-35 belong. Other cytokines whose biological activity is directly or indirectly related to IL-12 are IFN-γ, IL-4, and IL-10. The principal target cells and biological effects of IFN-γ are macrophages (classical activation and increased microbicidal functions) and T cells (Th1 differentiation), IL-4 T cells (Th2 differentiation and proliferation) and macrophages (alternative activation and inhibition of IFN-γ-mediated classical activation), and IL-10 macrophages and dendritic cells (inhibition of IL-12 expression) [1,2,3].

Corneal wound healing after stromal photoablation performed with LASIK (laser in situ keratomileusis) or PRK (photorefractive keratectomy) is a factor that affects the effectiveness and safety of these procedures. Reparation processes and the unpredictable nature of the corneal cell response directly correlate with clinical results as well as numerous complications. In addition to their positive effect in terms of correction of existing ametropia, both methods lead to surgically induced trauma. A complex cascade of cytokine-mediated cellular interactions is involved in response to trauma, such as intertwined interactions of epithelial, stromal, neural, lacrimal, and immune system cells. The interactions among these cells define the corneal response during wound healing and contribute to the regeneration and preservation of the anatomy and normal physiology of the cornea. These are important reasons to examine the participation of cytokines in the corneal wound healing process after stromal photoablation by the LASIK and PRK methods [4,5].

There is an increased production of the proinflammatory cytokines IL-1β and IL-8 during the acute phase of the inflammatory response in wound healing after LASIK treatment. Twenty-four hours after treatment, the concentration of IL-1β in tears is significantly higher after LASIK compared to PRK treatment. In addition, the acute phase of inflammation during corneal wound healing after PRK treatment is followed by an increased production of the proinflammatory cytokines TNF-α, IL-6, and IL-8. Twenty-four hours after treatment, there is a significantly larger concentration of IL-6 in the tears after PRK compared to LASIK treatment. Both PRK and LASIK are characterized by a significant inflammatory response [6].

Trauma caused by photoablation of the stroma results in a complex recovery process of the ocular surface (cornea and conjunctiva), but its mechanism and genetic control are not yet fully understood. The process integrates the functions of numerous cytokines, growth factors, and proteases produced by corneal epithelial cells, stromal keratocytes, the lacrimal gland, and inflammatory cells. The protein profiles of tears, including cytokine profiles, change dramatically after stromal photoablation. The following cytokines and growth factors are known to play a significant role in the recovery process: IL-1, IL-6, IL-8, TNF-a, PDGF-BB, TGF-β1, VEGF, HGF, and NGF [7,8]. The role of IL-1β and IL-10 in stimulating stromal cell apoptosis in postoperative recovery after transepithelial excimer laser treatment is shown by Cao et al. [9].

Knowing that IL-12 stimulates IFN-γ activity, reduces the inhibitory effect of IL-4 on IFN-γ, and that IL-10 inhibits IL-12 activity [10], the subject of interest was to examine the production and effect of IL-12, as a main representative of its family of heterodimeric cytokines, in the early phase of the immune response after excimer laser treatment, as well as study the relationship of this cytokine with IFN-γ, IL-4, and IL-10 in tears.

The aim of this research was to determine the concentrations of IL-12, IL-4, IL-10, and IFN-γ in tears undergoing different keratorefractive procedures and to gain insight into the role of IL-12 in postoperative inflammation.

## 2. Materials and Methods

A clinical, prospective cohort study was conducted in accordance with the Declaration of Helsinki. The dataset includes patients who underwent refractive surgery at the Military Medical Academy (MMA) in Belgrade, Serbia. The Ethics Committee of the MMA approved the prospective study. Patients were preoperatively assessed, and a refractive procedure was assigned according to standard medical indications. Specifically, LASIK was the preferred method, except for eyes with thin corneas (CCT < 500 µm or patients where the residual stromal bed would be < 300 µm), eyes with a flat (flat K < 41 D) or steep cornea (steep K > 48 D), deep set eyes or eyes with a small palpebral fissure, the presence of peripheral retinal degeneration (lattice or snail-track), or in patients who have a predisposition for contact injury [11]. Written informed consent was signed by all participants. Only myopic eyes with up to −3.0 D refractive spherical equivalent were selected in order to minimize variance in the energy used during the procedure. Other exclusion criteria for the study, in addition to the regular contraindications for the respective refractive procedures (including suspected keratoconus), were dry eye syndrome, Meibomian gland dysfunction, and a history of allergic conjunctivitis.

A topical anesthetic (4 mg/mL of oxybuprocaine) and one drop of a povidone-iodine 5% solution were administered before the surgery.

During the refractive procedure, a Wavelight Allegretto (400 Hz) excimer laser was used to perform photoablation in a 6.5 mm optical zone. By using this optical zone for all treatments, we again minimize the variance in the energy used during the procedure [12]. For LASIK, a Moria microkeratome (One Use-Plus SBK, Moria, France) was used to create a flap with a thickness of 130 µm. For PRK, the corneal epithelium was removed with a rotating brush (Amoils, Innovative Excimer Solutions, Inc., Toronto, ON, Canada).

No Mitomycin C was used during PRK since only eyes with small refractive errors were treated. After the PRK procedure, a therapeutic contact lens (Ciba Vision Night and Day, Ciba Vision, Duluth, GA, USA) was placed on the treated eye and removed on the 4th or 5th day after the intervention, when re-epithelialization of the cornea is achieved (as seen on a slit-lamp examination). After LASIK, no therapeutic contact lenses were used.

During the first 24 h post-intervention, the participants used only artificial tears with a sodium hyaluronate 1 mg/mL solution every hour and a Tobramycin 3 mg/mL solution 4 times per day during waking hours. The participants did not use any anti-inflammatory therapy 24 h post-intervention (neither corticosteroids nor NSAIDs). After the initial 24 h, i.e., after the third tear sample was collected, patients resumed standard therapy for their respective refractive procedures, including the use of anti-inflammatory therapy.

In the study, 35 patients were included, for a total of 68 eyes (for 2 of the patients, only 1 eye was operated on), of which 31 underwent LASIK and 37 underwent PRK. The PRK group included 19 patients, 14 of whom were male and 5 were female, whereas the LASIK group included 16 patients, 9 of whom were male and 7 were female. The average age of the participants was 34 (33.81 ± 6.52) years in the LASIK group and 33 (33.05 ± 6.11) years in the PRK group.

Three tear film samples were obtained from treated eyes: just before the procedure (t_0_), 1 h after the procedure (t_1_), and 24 h after the procedure (t_2_), for a total of 204 samples. Tear film samples were collected using a cellulose microsurgical sponge (MicroSponge™ regular tip, Alcon, Inc., Hünenberg, Switzerland) from the lower lateral tear meniscus with minimal irritation of the ocular surface and the edges of the eyelids, without (before) the use of anesthetics. After sampling, the tear fluid was separated using a centrifuge (MPW-350r; MED Instruments, Warsaw, Poland) at 13,000 RPM for 15 min at 4 °C into 0.5 mL of phosphate buffered saline (PBS). The samples were stored at −80 °C until analysis. This sampling method was described in Acera et al. [13].

The concentrations of IL-12 (referring to IL-12p70), IL-4, IL-10, and IFN-γ in the tear film samples were determined by flow cytometry using the Beckman Coulter FC 500 flow cytometer with CXP analysis software. A commercial test kit was used, the Human Th1/Th2 11-plex FlowCytomix Multiplex (Bender MedSystem, Vienna, Austria), intended for cytokine detection with a small sample volume.

Data analysis and visualization were conducted in Python 3 using the Anaconda distribution and included libraries. Shapiro-Wilk tests were used for all cytokine concentration values at t_0_, t_1_, and t_2_ in the LASIK and PRK groups to determine the type of distribution in order to decide the types of tests to be used. Wilcoxon signed-rank tests were used to compare the differences between t_0_ and t_1_, t_0_ and t_2_, and t_1_ and t_2_ for all cytokines in both LASIK and PRK groups. Mann-Whitney-U tests were used to compare the differences in the concentration of all cytokines between the two groups for t_0_, t_1_, and t_2_, respectively. Multiple linear regression was used to check if variations in IL-12 values could be explained by changes in the values of IL-10, IL-4, and IFN-γ.

## 3. Results

**Figure 1:**
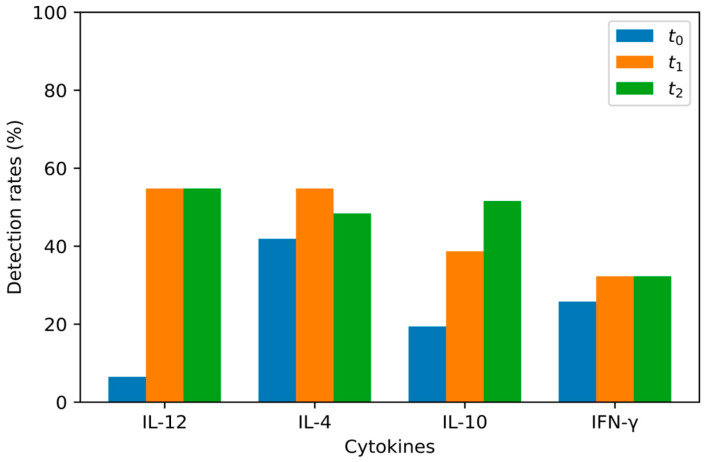
Detection rates for IL-12 t_0–2_, IL-4 t_0–2_, IL-10 t_0–2_, and IFN-γ t_0–2_ in the LASIK group.

**Figure 2:**
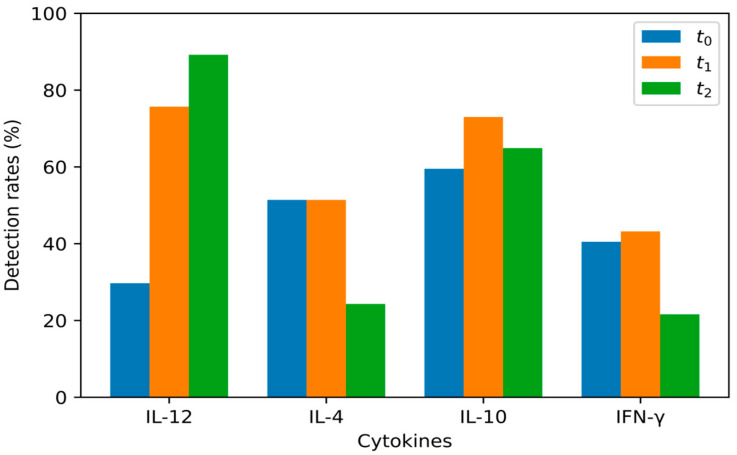
Detection rates for IL-12 t_0–2_, IL-4 t_0–2_, IL-10 t_0–2_, and IFN-γ t_0–2_ in the PRK group.

Table 1, Table 2, Table 3, Table 4 and Table 5 show the descriptive statistics for our results, specifically for the mean values, median values, and detection rates, as well as the lowest and highest detected values. Figure 1 and Figure 2 show the detection rates at t_0_, t_1_ and t_2_ for LASIK and PRK respectively. Table 6, Table 7, Table 8 and Table 9 show the *p* values for the statistical analyses of the results shown in Table 1, Table 2, Table 3, Table 4 and Table 5. The test power for each performed statistical test in Table 6, Table 7, Table 8, Table 9 and Table 10 can be found in Table 11.

Shapiro-Wilk tests on all data subsets yielded a *p* < 0.005, which suggests that the data is not normally distributed. Therefore, nonparametric tests were used for analyzing the data.

For IL-12, statistically significant differences were noted between t_0_ and t_1_, as well as t_0_ and t_2_, in the LASIK and PRK groups (*p* < 0.001). However, between t_1_ and t_2_, while a statistically significant difference was noted in the PRK group (*p* < 0.005), no difference was found in the LASIK group.

For IL-4, no differences were found between t_0_ and t_1_ in either group. A statistically significant difference between t_0_ and t_2_ and between t_1_ and t_2_ was found in the PRK group (*p* < 0.05), but not in the LASIK group.

For IL-10, between t_0_ and t_1_, there was a statistically significant difference in the LASIK group (*p* < 0.05) and the PRK group (*p* < 0.05). Between t_0_ and t_2_, a difference was noted in the LASIK group (*p* < 0.05), but not in the PRK group. No differences were found between t_1_ and t_2_ in any of the groups.

For IFN-γ, no statistically significant differences were found between t_0_, t_1_, and t_2_ in any of the groups.

For IL-12, statistically significant differences were observed in t_0_ (*p* < 0.05), t_1_ (*p* < 0.05), and t_2_ (*p* <0.001). For IL-4, a statistically significant difference between the two groups was found only for t_2_ (*p* < 0.05). For IFN-γ, no differences were observed between the two groups for t_0_, t_1_, and t_2_. For IL-10, a statistically significant difference between the LASIK and PRK groups was found for t_0_ (*p* < 0.005) and t_1_ (*p* < 0.005), while no difference was found for t_2_.

**Figure 3:**
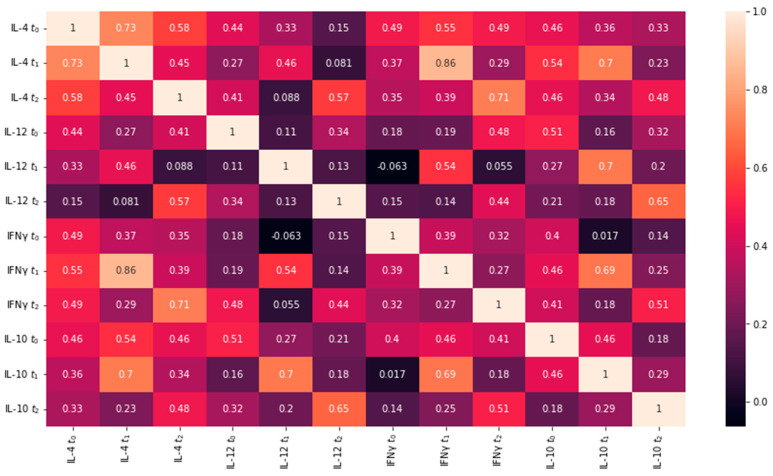
Collinearity matrix for data from the LASIK group. Each value represents the Spearman correlation coefficient (r) between the two variables, displayed as a heat map.

**Figure 4:**
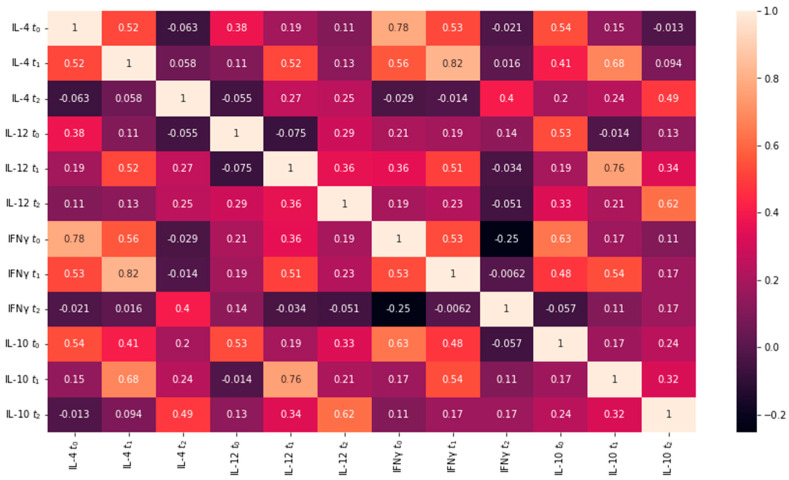
Collinearity matrix for data from the PRK group. Each value represents the Spearman correlation coefficient (r) between the two variables, displayed as a heat map.

Multiple linear regression was used to test if the measured IL-4, IL-10, and IFN-γ values at t_0_, t_1_, and t_2_ predicted IL-12 values at t_0_, t_1_, and t_2_ for both LASIK and PRK groups. Only values measured at t_0_ were used in the regression model for IL-12 t_0_, and only values measured at t_0_ and t_1_ were used in the regression model for IL-12 t_1_. Figure 3 and Figure 4 show the collinearity matrices for the LASIK and PRK groups respectively.

### 3.1. Results of Multiple Linear Regression Analysis of IL-12 in Samples Taken before the Procedure (t_0_)

Multiple linear regression was used to test if IL-4 t_0_, IL-10 t_0_, and IFN-γ t_0_ significantly predicted IL-12 values for t_0_ for eyes in the LASIK group. The overall regression was statistically significant (F = 5.357, *p* = 0.005) with a coefficient of determination of R^2^ = 0.373 (Adjusted R^2^ = 0.303). It was found that IL-4 t_0_ (β = 0.2173, *p* = 0.049) and IL-10 t_0_ (β = 0.2894, *p* = 0.011) significantly predicted IL-12 values for t_0_ in the LASIK group.

Multiple linear regression was used to test if IL-4 t_0_, IL-10 t_0_, and IFN-γ t_0_ significantly predicted IL-12 values for t_0_ for eyes in the PRK group. The overall regression was statistically significant (F = 4.421, *p* = 0.01) with a coefficient of determination of R^2^ = 0.287 (Adjusted R^2^ = 0.222). It was found that IL-4 t_0_ (β = 0.609, *p* = 0.027) and IFN-γ t_0_ (β = −0.411, *p* = 0.049) significantly predicted IL-12 values for t_0_ in the PRK group.

The results of our study show that in the LASIK group at t_0_, there is a positive correlation between IL-4 and IL-10 concentrations with IL-12 concentrations in tear film. In the PRK group at t_0_, there is a positive correlation between IL-4 and IL-12 concentrations, as well as a negative correlation between IFN-γ and IL-12. It is important to note that these are results at t_0_, which is the normal physiological state of the eyes before undergoing any sort of treatment.

### 3.2. Results of Multiple Linear Regression Analysis of IL-12 in Samples Taken One Hour Post-Intervention (t_1_)

Multiple linear regression was used to test if IL-4 t_0–1_, IL-10 t_0–1_, and IFN-γ t_0–1_ significantly predicted IL-12 values for t_1_ for eyes in the LASIK group. The overall regression was statistically significant (F = 2.637, *p* = 0.041) with a coefficient of determination of R^2^ = 0.397 (Adjusted R^2^ = 0.247). No statistically significant correlation was found between individual variables and IL-12 concentration.

Multiple linear regression was used to test if IL-4 t_0–1_, IL-10 t_0–1_, and IFN-γ t_0–1_ significantly predicted IL-12 values for t_1_ for eyes in the PRK group. The overall regression was statistically significant (F = 5.762, *p* = 0.000) with a coefficient of determination of R^2^ = 0.535 (Adjusted R^2^ = 0.442). It was found that IL-10 t_1_ (β = 0.4396, *p* = 0.000) significantly predicted IL-12 values for t_1_.

The results for the LASIK group at t_1_ show that there is no statistically significant correlation between IL-10, IL-4, and IFN-γ at t_0_ and t_1_ with IL-12 at t_1_. The results for the PRK group at t_1_ show a positive correlation between IL-10 t_1_ values and IL-12 t_1_ values.

### 3.3. Results of Multiple Linear Regression Analysis of IL-12 in Samples Taken 24 Hours Post-Intervention (t_2_)

Multiple linear regression was used to test if IL-4 t_0–2_, IL-10 t_0–2_, and IFN-γ t_0–2_ significantly predicted IL-12 values for t_2_ for eyes in the LASIK group. The overall regression was statistically significant (F = 2.922, *p* = 0.000) with a coefficient of determination of R^2^ = 0.556 (Adjusted R^2^ = 0.366). No statistically significant correlation was found between individual variables and IL-12 concentration.

Multiple linear regression was used to test if IL-4 t_0–2_, IL-10 t_0–2_, and IFN-γ t_0–2_ significantly predicted IL-12 values for t_2_ for eyes in the PRK group. The overall regression was not statistically significant (F = 1.722, *p* = 0.132).

No statistically significant correlation was found between IL-10, IL-4, and IFN-γ values at t_0_, t_1_, and t_2_ with IL-12 t_2_ values.

## 4. Discussion

The first phase of the recovery process after photoablation is the removal of damaged tissue. Next follows a gradual replacement of damaged tissue with healthy tissue and, eventually, control and prevention of uncontrolled growth and proliferation. The goal is to establish normal corneal microanatomy and, with that, corneal transparency [8].

In the present study, the roles of IL-12, IL-4, IL-10, and IFN-γ in the early phase of the immune response after excimer laser treatment were examined, and by analyzing these cytokines, a potential explanation was provided regarding the role of IL-12 in connection to the inflammatory and anti-inflammatory responses after treatment. The initial hypothesis was that in the first 24 h, the inflammatory response would be greater after PRK but that the anti-inflammatory response would be greater after LASIK.

During PRK, the corneal epithelium was removed using a rotational brush (mechanical epithelial debridement), and this method is the standard way of epithelium removal at the MMA hospital. The corneal epithelium may also be removed by applying an alcohol solution (alcohol-assisted epithelial debridement). Different inflammatory dynamics may be expected when using other methods of epithelium removal [14], and future studies should address this issue.

For each analyzed cytokine, certain measurements at certain time points could not detect cytokine concentrations (Table 3). This may be due to the existence of an unknown detection threshold for each cytokine that is not specified in the test kit manuals. Table 4 shows the lowest detected values for every cytokine at t_0_, t_1_, and t_2_ for both LASIK and PRK groups, and it is shown that for each cytokine, the lowest detected values are similar, no matter the sampling time. Detection rates for the samples gathered at t_0_ of IL-12, IL-4, IL-10, and IFN-γ were 6.5%, 41.9%, 19.4%, and 25.8%, respectively, in the LASIK group, and 29.7%, 51.4%, 59.5%, and 40.5%, respectively, in the PRK group. LaFrance et al. compared different instruments and reagents used in the detection of cytokines in the tear films of healthy people. The instruments used in this study are all “cytometric bead-based assays,” and the results show detectability rates for IL-12, IL-4, IL-10, and IFN-γ of 73%, 97%, 79%, and 91%, respectively [15].

The results showed that there is a statistically significant difference between the procedures in IL-10 values one hour post-intervention (t_1_), with the median values, mean values, and detection rates of IL-10 being larger in the PRK group than in the LASIK group.

The data showed a statistically significant difference between the procedures in IL-4 concentrations 24 h post-intervention, with the median values and detection rates of IL-4 being larger in the LASIK group than in the PRK group. No differences were found in IL-4 values one hour post-intervention, so it can be concluded that a difference in the IL-4 responses only exists in the late postoperative stage (24 h post-intervention).

The results of the regression analyses determined that in the LASIK group at t_0_, a positive correlation is shown between IL-12 values and IL-4 and IL-10 values, respectively. In the PRK group at t_0_, there is a positive correlation between IL-12 and IL-4 values and a negative correlation between IL-12 and IFN-γ values. It is important to note that these results represent the normal physiological state of the tear film.

Multiple linear regression analysis revealed that for the LASIK group, there is no statistically significant correlation between IL-12 values at t_0–1_ and IL-10, IL-4, and IFN-γ values at t_0–1_. The results for the PRK group showed that at t_1_, there is a positive correlation between IL-10 t_1_ and IL-12 t_1_. Because this correlation was seen in the PRK group but not in the LASIK group, it may suggest that IL-10 plays a larger role in the regulation of the early postoperative immune response (one hour after surgery) after PRK than after LASIK.

The results of the linear regression for both LASIK and PRK groups do not show a statistically significant correlation between IL-10 t_0–2_, IL-4 t_0–2_, and IFN-γ t_0–2_ with IL-12 t_2_.

In the PRK group at t_1_, there is a statistically significant correlation between IL-10 t_1_ and the values of IL-12 t_1_, a result that does not persist at t_2_. The result suggests that IL-10 may play a larger role in the regulation of IL-12 in the early (one hour post-intervention) postoperative immune response after PRK. This was confirmed by the results in Table 10. The results for IL-4, where a difference was only detected at t_2_, suggest that IL-4 plays a larger role in the late (24 h post-intervention) postoperative immune response after LASIK compared to PRK.

IL-12 values were detected in 6.5% of samples taken before LASIK and 29.7% of samples taken before PRK (an average of 19.1% for all samples). One hour post-intervention, it was detected in 54.8% of samples in the LASIK group and 75.7% of samples in the PRK group (an average of 66.2% for all samples). After 24 h post-intervention, IL-12 was detected in 54.8% of samples from eyes in the LASIK group and 89.2% of samples from eyes in the PRK group (an average of 73.5% for all samples).

The results for IL-12 in tears before any keratorefractive procedure can be explained by its role in innate immune defense through the complement system. IL-12 is key in controlling the activity of macrophages, granulocytes, NK, and NKT cells, as well as the late activation of T-cells. The production of biologically significant concentrations of IL-12 in tears would allow for controlled activation of phagocytic cells to clear debris during everyday activities without causing an uncontrolled inflammation cascade that would lead to opacities in the optic media.

The majority of corneal dendritic cells, which produce IL-12, are found in the inferior peripheral cornea (three times more than in the central cornea) [16]. The statistically significant difference in IL-12 concentration in tears after PRK compared to LASIK can be explained by the increased activity of macrophages and dendritic cells after PRK, which may be one of the factors responsible for the appearance of corneal haze. Therefore, it can be hypothesized that corneal haze appears when a certain unknown threshold in the activation of these cells is passed. If this were to be true, high-risk patients (those in which an overproduction of cytokines occurs) could be treated locally with a specific anti-cytokine antibody cocktail as haze prophylaxis. Future studies may develop more specific methods to identify these high-risk patients. Leonardi et al. showed a significant increase in IL-12 in tears one hour after LASIK in 10 out of 15 patients (66.7%). In corneal fibroblast cultures, IL-12 was not detected before nor after undergoing photoablation by excimer laser [17]. Alio et al. explain that the increase determined in the study by Leonardi et al. is probably a consequence of corneal dendritic cell stimulation [18].

The cascade of corneal wound healing after the PRK and LASIK methods is initiated by epithelial injury, and the intensity of the response is proportional to the size of the epithelial damage. For this reason, many of the events involved in corneal wound healing are more intense after performing PRK compared to the LASIK method [19]. Injured epithelial cells release more pro-inflammatory mediators, which bind to specific receptors on stromal cells and modulate processes such as keratocyte apoptosis, fibroblast proliferation, myofibroblast formation, and inflammatory cell influx [20]. After the LASIK method, the trauma to the epithelium is significantly smaller compared to the PRK method and tends to be confined to the edge of the flap. The clinical consequences of a more intensive response during corneal wound healing include slower vision rehabilitation, more pronounced hyperplasia of the epithelium, remodeling of the stroma that can lead to regression, and more pronounced formation of myofibroblasts, followed by the appearance of corneal haze [21,22]. Topical application of moxifloxacin, a fourth-generation fluoroquinolone antibiotic, affects fibroblasts, decreasing their tendency to transform into myofibroblasts, whose appearance is related to corneal haze formation [23]. Moxifloxacin inhibits human corneal fibroblast migration by suppressing IL-12 secretion [24]. In our study, we obtained higher levels of IL-12 in tears after PRK compared to the LASIK method, signifying that the pro-inflammatory response is more pronounced after PRK compared to LASIK. For this reason, it might be recommended to use moxifloxacin drops in local therapy during the first postoperative days after PRK, at least until re-epithelialization of the cornea is achieved. This might be a good alternative to the use of mitomycin-C immediately after photoablation of the stroma since there are studies showing a certain degree of toxicity of this agent to corneal endothelial cells [25,26].

IL-4 values were detected in 41.9% of samples taken before LASIK and 51.4% of samples taken before PRK (an average of 47.1% for all samples). One hour post-intervention, it was detected in 54.8% of samples in the LASIK group and 51.4% of samples in the PRK group (an average of 52.3% for all samples). After 24 h post-intervention, IL-4 was detected in 48.4% of samples in the LASIK group and 24.3% of samples in the PRK group (an average of 35.3% of all samples).

One hour post-intervention, there was no statistically significant difference in IL-4 concentrations between the LASIK and PRK groups. A statistically significant difference (*p* < 0.05) in IL-4 between the LASIK and PRK groups was found only in the samples taken 24 h post-intervention, with a significant drop in IL-4 concentration in the PRK group. This could be explained by the use of a vacuum suction ring for fixation of the eye only during LASIK, causing mechanical irritation of the conjunctiva. Since mast cells in the conjunctiva are a primary source of IL-4, it may cause the activation of an IL-4 cascade and increased values. In the PRK group, there was a significant decrease in IL-4 concentrations 24 h post-intervention, accompanied by an increase in IL-12 concentrations, suggesting that the late pro-inflammatory response (24 h post-intervention) is more pronounced after PRK. Before treatment, Leonardi et al. detected IL-4 at sub-threshold values (5 pg/mL) in 11 out of 15 patients; after LASIK, they found no changes in the concentration of this cytokine. Furthermore, they did not detect IL-4 in corneal fibroblast cultures, neither before nor after exposing the cultures to photoablation by excimer laser [17]. In a review, Leonardi discussed mediators of allergic reactions in tears, reporting that studies have shown that cytokines belonging to the group of cytokines produced by Th2 cells (IL-4, IL-5, and IL-13) are significantly increased in the tear film of eyes with atopic diseases compared to the tears of healthy eyes [27]. IL-4 is produced by T lymphocytes, basophils, and mast cells, and it has an anti-inflammatory effect by inhibiting TNF-α, IL-1, and IL-6 [28]. Ellenberg et al. [29] showed that IL-1 and IL-8 are key in the modulation of the corneal response by inducing angiogenesis. Volpert et al. [30] show that IL-4 can inhibit angiogenesis.

IL-10 values were detected in 19.4% of samples taken before LASIK and 59.5% of samples taken before PRK (an average of 41.1% for all samples). One hour post-intervention, it was detected in 38.7% of samples in the LASIK group and 73% of samples in the PRK group (an average of 57.3% for all samples). After 24 h post-intervention, IL-10 was detected in 51.6% of samples in the LASIK group and 64.9% of samples in the PRK group (an average of 58.9% of all samples). The IL-10 concentrations differed significantly between the LASIK and PRK groups even before the surgery. This might be due to physiological variations in IL-10 concentrations because IL-10, with its effects, maintains a non-inflamed environment during normal function, preventing any minor causes of inflammation that may cause opacification of the ocular media during daily activities. There is also a statistically significant difference in IL-10 values between the LASIK and PRK groups one hour post-intervention. In both the LASIK and PRK groups, there is an increase in IL-10 concentration one hour post-intervention, probably as a response to an increase in IL-12, which can be found in both groups. However, the increase in IL-10 persists 24 h post-intervention in the LASIK group but not in the PRK group. This is accompanied by a decrease in IL-12 values in the LASIK group 24 h post-intervention, although not a statistically significant one. This suggests that the late anti-inflammatory effects of IL-10 are more pronounced after LASIK. Leonardi et al. did not detect IL-10 in tear samples before or after LASIK treatment, nor was it found in corneal fibroblast cultures before or after photoablation by excimer laser [17]. IL-10 is produced by macrophages and T cells, and it has an anti-inflammatory effect by inhibiting the production of TNF-α, IL-1, and IL-6 [28].

IFN-γ values were detected in 25.8% of samples taken before LASIK and 40.5% of samples taken before PRK (an average of 33.8% for all samples). One hour post-intervention, it was detected in 32.3% of samples in the LASIK group and 43.2% of samples in the PRK group (an average of 38.2% for all samples). After 24 h post-intervention, IFN-γ was detected in 32.3% of samples in the LASIK group and 21.6% of samples in the PRK group (an average of 26.5% for all samples). No statistically significant differences were found in IFN-γ concentrations between tear samples taken before, one hour after, and 24 h after LASIK and PRK treatment. The results matched the data from Leonardi et al. In their study, they detected subthreshold IFN-γ values in 11 out of 15 patients without an increase after LASIK. They also did not detect IFN-γ in corneal fibroblast cultures before or after exposure to photoablation by excimer laser [17]. IFN-γ is produced by NK cells and activated CD4+ Th1 cells. The IFN-γ receptor is found in the conjunctival and corneal epithelium [31]. It has a pleiotropic effect, and its main function is the activation of macrophages in both the innate and acquired cellular immune responses. During both LASIK and PRK treatments, there is iatrogenic damage to the corneal epithelium. A similar damage to the epithelium occurs when performing the corneal cross-linking (CXL) procedure. Kolozsvari et al. examined the effects of CXL, which is used to slow the progression of keratoconus, on inflammatory mediators in the tear film and their relation to changes in corneal architecture. Tears were sampled from 26 eyes in 23 patients before CXL and 12 months after the procedure, and their concentrations were determined using cytometric bead arrays. Four days after the procedure, there was an increase in the concentrations of IL-6 and IL-8 and a significant decrease in IL-13, IL-17A, IFN-γ, CCL5, MMP-13, EGF, NGF, and PAI-1 when compared to baseline values before the intervention [32].

In our study, there were no postoperative complications in terms of hypercorrection, hypocorrection, regression, or haze. Their absence is a consequence of proper preoperative evaluation, regular postoperative monitoring, and the diopter value that needed to be corrected, up to −3.0 D refractive spherical equivalent, so there was no need for the intraoperative use of mitomycin-C. The absence of haze can also be attributed to the regular implementation of prescribed postoperative therapy. Namely, after the PRK procedure, the patients used corticosteroid drops (the first-month drops containing dexamethasone 0.1% and the second- and third-month drops containing prednisolone 0.5%). Kaji et al. show in their study that local application of corticosteroids (betamethasone) effectively suppresses the appearance of haze after excimer laser keratectomy [33].

The present study, taking into account the results of previous studies, indicates the possibility of using pharmacological agents whose application in high-risk patients would have a positive effect on preventing the possible occurrence of corneal haze. In this sense, the postoperative topical application of moxifloxacin, especially after PRK, would be recommended, though more research is needed. It might also be beneficial to apply specific anti-cytokine antibodies to prevent the appearance of haze instead of mitomycin-C, whose application can be accompanied by the appearance of adverse reactions, most notably a decrease in the number of endothelial cells [26]. With the postoperative determination of cytokine levels in tears (IL-1β, IL-4, IL-6, IL-8, IL-10, IL-12, and TNF-α), it would be possible to identify patients who are at high risk of developing haze, and timely, topical application of specific anti-cytokine antibodies would prevent its occurrence.

One limitation of the study was the inability to monitor the long-term (after 24 h) levels of cytokines in the tear film due to the need for anti-inflammatory therapy after the procedures. It was also impossible to examine the levels of cytokines after keratorefractive procedures in patients with a larger refractive error since our study was limited to nearsighted eyes with up to −3.0 D refractive spherical equivalent.

## 5. Conclusions

In conclusion, there is no difference in IL-4 concentrations one hour after treatment between LASIK and PRK, whereas there is a significant decrease in IL-4 concentrations 24 h after PRK but not after LASIK. In both LASIK and PRK groups, there is an increase in IL-10 values one hour after treatment, accompanied by an increase in IL-12, which can be found one hour after both types of surgery. However, the increase in IL-10 persists 24 h after LASIK but not after PRK, which leads to an increase in IL-12 24 h after PRK. This shows that 24 h after surgery, the pro-inflammatory response is more pronounced after PRK, while the anti-inflammatory response is more pronounced after LASIK.

## Figures and Tables

**Table 1:** The mean values for IL-12 t_0–2_, IL-4 t_0–2_, IL-10 t_0–2_, and IFN-γ t_0–2_ for the LASIK and PRK groups.

Mean Values (pg/mL)	LASIK Group, *n* = 31			PRK Group, *n* = 37		
	t_0_	t_1_	t_2_	t_0_	t_1_	t_2_
IL-12	5.796	50.952	49.911	26.565	72.522	93.803
IL-4	24.478	31.529	26.373	26.375	31.261	8.464
IL-10	17.459	43.572	46.970	55.687	89.299	81.704
IFN-γ	22.989	31.923	31.518	36.713	46.804	21.622

**Table 2:** The median values for IL-12 t_0–2_, IL-4 t_0–2_, IL-10 t_0–2_, and IFN-γ t_0–2_ for the LASIK and PRK groups.

Median Values (pg/mL)	LASIK Group, *n* = 31			PRK Group, *n* = 37		
	t_0_	t_1_	t_2_	t_0_	t_1_	t_2_
IL-12	0	86.44	86.26	0	91.05	99.1
IL-4	0.0	6.31	0	4.17	2.07	0
IL-10	0	0	75.64	82.99	100.34	86.47
IFN-γ	0	0	0	0	0	0

**Table 3:** Detection rates for IL-12 t_0–2_, IL-4 t_0–2_, IL-10 t_0–2_, and IFN-γ t_0–2_ for both LASIK and PRK groups.

Detection Rates (%)	LASIK Group, *n* = 31			PRK Group, *n* = 37		
	t_0_	t_1_	t_2_	t_0_	t_1_	t_2_
IL-12	6.5%	54.8%	54.8%	29.7%	75.7%	89.2%
IL-4	41.9%	54.8%	48.4%	51.4%	51.4%	24.3%
IL-10	19.4%	38.7%	51.6%	59.5%	73.0%	64.9%
IFN-γ	25.8%	32.3%	32.3%	40.5%	43.2%	21.6%

**Table 4:** Lowest detected values for IL-12 t_0–2_, IL-4 t_0–2_, IL-10 t_0–2_, and IFN-γ t_0–2_ for both LASIK and PRK groups.

Lowest Detected Values (pg/mL)	LASIK Group, *n* = 31			PRK Group, *n* = 37		
	t_0_	t_1_	t_2_	t_0_	t_1_	t_2_
IL-12	88.64	86.15	85.91	86.21	87.4	86.14
IL-4	7.4	2.59	7.58	4.17	2.07	1.58
IL-10	79.35	80.08	75.64	77.19	79.35	70.53
IFN-γ	70.84	76.45	75.02	70.33	74.17	71.6

**Table 5:** Highest detected values for IL-12 t_0–2_, IL-4 t_0–2_, IL-10 t_0–2_, and IFN-γ t_0–2_ for both LASIK and PRK groups.

Highest Detected Values (pg/mL)	LASIK Group, *n* = 31			PRK Group, *n* = 37		
	t_0_	t_1_	t_2_	t_0_	t_1_	t_2_
IL-12	91.05	133.53	109.99	100.86	113.31	147.43
IL-4	87.81	96.71	97.31	90.14	101.07	96.31
IL-10	103.80	198.33	121.84	148.85	215.52	279.06
IFN-γ	123.87	204.33	157.75	155.51	172.54	202.61

**Table 6:** Differences in IL-12 concentrations between t_0_ and t_1_, t_0_ and t_2_, and t_1_ and t_2_.

IL-12	LASIK Group	PRK Group
t_0_ and t_1_	*p* < 0.001	*p* < 0.001
t_0_ and t_2_	*p* < 0.001	*p* < 0.001
t_1_ and t_2_	*p* = 0.775	*p* < 0.005

**Table 7:** Differences in IL-4 concentrations between t_0_ and t_1_, t_0_ and t_2_, and t_1_ and t_2_.

IL-4	LASIK Group	PRK Group
t_0_ and t_1_	*p* = 0.139	*p* = 0.638
t_0_ and t_2_	*p* = 0.421	*p* < 0.05
t_1_ and t_2_	*p* = 0.476	*p* < 0.05

**Table 8:** Differences in IL-10 concentrations between t_0_ and t_1_, t_0_ and t_2_, and t_1_ and t_2_.

IL-10	LASIK Group	PRK Group
t_0_ and t_1_	*p* < 0.05	*p* < 0.05
t_0_ and t_2_	*p* < 0.05	*p* = 0.081
t_1_ and t_2_	*p* = 0.822	*p* = 0.586

**Table 9:** Differences in IFN-γ concentrations between t_0_ and t_1_, t_0_ and t_2_, and t_1_ and t_2_.

IFN-γ	LASIK Group	PRK Group
t_0_ and t_1_	*p* = 0.382	*p* = 0.135
t_0_ and t_2_	*p* = 0.470	*p* = 0.223
t_1_ and t_2_	*p* = 0.868	*p* = 0.067

**Table 10:** Differences in cytokine concentrations between the LASIK and PRK groups for t_0_, t_1_, and t_2_.

LASIK vs. PRK	IL-12	IL-4	IFN-γ	IL-10
t_0_	*p* < 0.05	*p* = 0.562	*p* = 0.220	*p* < 0.005
t_1_	*p* < 0.05	*p* = 0.990	*p* = 0.240	*p* < 0.005
t_2_	*p* < 0.001	*p* < 0.05	*p* = 0.314	*p* = 0.052

**Table 11:** Study power of each performed statistical test.

Statistical Test		IL-12	IL-10	IL-4	IFN-γ
Mann-Whitney-U tests	t_0_	0.74	0.96	0.04	0.24
	t_1_	0.49	0.86	0.02	0.2
	t_2_	0.99	0.65	0.62	0.13
Wilcoxon signed-rank tests (LASIK)	t_0_ and t_1_	0.99	0.53	0.10	0.11
	t_0_ and t_2_	0.99	0.78	0.03	0.11
	t_1_ and t_2_	0.03	0.04	0.07	0.02
Wilcoxon signed-rank tests (PRK)	t_0_ and t_1_	0.99	0.73	0.07	0.12
	t_0_ and t_2_	1	0.43	0.69	0.28
	t_1_ and t_2_	0.64	0.06	0.82	0.54

## Data Availability

The data presented in this study are available on request from the first and corresponding author, in particular the datasets are archived in the clinic where the participants were treated. The data are not publicly available as they contain information that could compromise the privacy of the participants.
